# Long-term physical, mental and social health effects of COVID-19 in the pediatric population: a scoping review

**DOI:** 10.1007/s12519-022-00515-7

**Published:** 2022-02-03

**Authors:** Madeline Borel, Luyu Xie, Olivia Kapera, Adrian Mihalcea, Jeffrey Kahn, Sarah E. Messiah

**Affiliations:** 1 School of Public Health, University of Texas Health Science Center, Dallas Campus, 2777 N Stemmons Fwy, Dallas, TX 75207 USA; 2 Center for Pediatric Population Health, University of Texas Health School of Public Health and Children’s Health System of Texas, Dallas, TX USA; 3 School of Public Health, University of Texas Health Science Center, Austin Campus, Austin, TX USA; 4 School of Public Health, University of Texas Health Science Center, Houston Campus, Houston, TX USA; 5 Department of Pediatrics, University of Texas Southwestern Medical Center, Dallas, TX USA; 6 Children’s Health System of Texas, Dallas, TX USA

**Keywords:** Adolescents, Children, Coronavirus disease 2019 (COVID-19), Long-COVID symptoms

## Abstract

**Background:**

The majority of coronavirus disease 2019 (COVID-19) symptom presentations in adults and children appear to run their course within a couple of weeks. However, a subgroup of adults has started to emerge with effects lasting several months or more after initial infection, which raises questions about the long-term physical, mental and social health effects of COVID-19 in the pediatric population. The purpose of this review was to determine these impacts well into the second year of the pandemic.

**Methods:**

A search was conducted using PubMed, Web of Science, Science Direct, and Cochrane between 11/1/2019 and 9/1/2021. Search inclusion criteria were as follows: (1) COVID-19 illness and symptoms in children; (2) severe acute respiratory syndrome coronavirus 2 in children; (3) English language; and (4) human studies only.

**Results:**

The few studies that have documented long-term physical symptoms in children show that fatigue, difficulty in concentrating (brain fog), sleep disturbances, and sensory problems are the most reported outcomes. Most studies examining the impact of COVID-19 in pediatric populations have focused on initial clinical presentation, and symptoms, which are similar to those in adult populations. In addition, COVID-19 has had a moderate impact on children and adolescents’ social environment, which may exacerbate current and future physiological, psychological, behavioral, and academic outcomes.

**Conclusions:**

There are limited studies reporting long physical symptoms of COVID-19 in the pediatric population. However, pediatric COVID-19 cases are underreported due to low rates of testing and symptomatic infection, which calls for more longitudinal studies. Children who have experienced COVID-19 illness should be monitored for long physiological, psychological, behavioral, and academic outcomes.

## Introduction

Severe acute respiratory syndrome coronavirus 2 (SARS-CoV-2) causes the novel coronavirus disease 2019 (COVID-19) and as of September 9, 2021, there have been 5.3 million reported cases among children in the United States [1]. Early studies from China [2–9] and Europe [10] have shown that COVID-19 is generally a mild disease in children, including infants. In the U.S., and globally, fewer cases of COVID-19 have been reported in children (age 0–17 years) compared with adults [1, 7] but recent data suggest the delta (B.1.617.2) variant is more transmissible among children compared to the alpha (B.1.1.7) variant. Specifically, while children comprise 22% of the U.S. population (novel coronavirus), recent data show that 15.5% of all cases of COVID-19 reported to the Centers for Disease Control and Prevention were among children [3]. Indeed, the true incidence of SARS-CoV-2 infection in children is not known due to lack of widespread testing and the prioritization of testing for adults and those with severe illness. In addition, hospitalization rates in children have remained significantly lower than adult rates suggesting that children may have less severe illness from COVID-19 compared to adults [9]. However, a small proportion of children develop severe disease requiring intensive care unit (ICU) admission and prolonged ventilation [11], although fatal outcome is rare. In addition, reports of a novel Kawasaki disease-like multisystem inflammatory syndrome (MIS-C) necessitate continued surveillance in pediatric patients [12–15]. This syndrome has also been reported as pediatric inflammatory multisystem syndrome temporally associated with SARS-CoV-2 (PIMS-TS) [16, 17].

Common acute symptoms of COVID-19 disease in both adults and children include fever, cough, shortness of breath, chills, muscle pain, headache, sore throat, and loss of taste or smell. The most common physical symptoms reported by adults after a SARS-CoV-2 infection are fatigue/lethargy and shortness of breath, with on average one-third reporting at least one persistent symptom months after recovery [18, 19]. Most patients recover within 2 weeks of initial symptoms. However, a subgroup of adults has been documented to have longer-lasting symptoms, often termed “long” or “long haulers”. One recent study has reported similar long symptoms in children (≤ 18 years old) previously diagnosed with COVID-19 from the largest hospital in Rome [20]. A questionnaire was delivered by two pediatricians, either online or during outpatient visit, between September 1st, 2020 and January 1st, 2021 to the children’s caregivers. Symptoms frequently reported up to 120 days after infection included muscle and joint pain, headache, insomnia, respiratory issues, and palpitations [20]. While this study did provide some insight into long-COVID-19 symptoms, the findings were limited by a single-center design and relatively small sample size.

The COVID-19 pandemic and its associated mitigation strategies are expected to have significant psychosocial, behavioral, socioeconomic, and health impacts, which are exacerbated in populations that experience health disparities and other vulnerable groups [21–23]. Pediatric populations experiencing health disparities prior to the COVID-19 pandemic are at increased risk of infection and other COVID-19 related consequences (e.g., prolonged school closings, low resources to support online learning, parent job loss, high prevalence of community morbidity and mortality due to COVID-19) [24, 25]. Preliminary reports in the U.S. point consistently to disparities by race and ethnicity, with African Americans, Hispanics/Latinos, American Indians/Alaska Natives, and Native Hawaiians/Other Pacific Islanders experiencing a greater COVID-19 burden than non-Hispanic White populations [26]. Reports by geographic locations indicate that cases are substantially greater in economically disadvantaged census tracts [1, 27]. These long-term effects, even if only mild in severity, can have a detrimental impact on a person’s overall quality of life [28]. Therefore, the purpose of this review is to gather evidence on the current state of knowledge of potential long symptoms and consequences of COVID-19 in the pediatric population including physical, mental, behavioral, and social health, academic, and quality-of-life outcomes.

## Methods

PRISMA guidelines were used as the search framework. A comprehensive search was completed via PubMed, Web of Science, Science Direct, medRxiv, and Cochrane with the following search terms: multisystem inflammatory syndrome or MIS-C, pediatric inflammatory multisystem syndrome temporally associated with SARS-CoV-2 or PIMS-TS, COVID-19 and/or SARS-CoV-2, and children, adolescent, adolescence, or pediatric. PubMed filters applied: abstract, clinical study, clinical trial, comparative study, controlled clinical trial, journal article, meta-analysis, observational study, randomized controlled trial, review, systematic review, humans, child: birth–18 years, newborn: birth–1 month, infant: birth–23 months, infant: 1–23 months, preschool child: 2–5 years, child: 6–12 years, adolescent: 13–18 years, from 11/1/2019 to 9/1/2021.

Studies were stratified by study setting and patient population type. Group A consisted of articles specifically mentioning MIS-C and/or PIMS-TS in their title, and/or their primary population diagnosed as MIS-C and/or PIMS-TS patients. Group B was defined by studies that took place within a hospital and/or participants having been studied for COVID-19 infection in any hospital department such as the emergency department, neonatal ICU, pediatric ICU, or ICU. If a population also included outpatients or was any study conducted outside of a hospital setting, then they were placed in Group C (non-hospital). After removing duplicates and reading title and abstract, the number of articles was reported by articles found, those selected for literature review, and those specifically pertaining to or considered as relating to long-term effects, for primary review.

### Permission to reproduce material from other sources

This study did not reproduce any material from other sources.

## Results

The total number of articles selected for review was 130, with 34 deemed relevant or directly pertaining to long-term effects following a COVID-19 infection and/or effects of the COVID-19 pandemic in the pediatric population. In general, inclusion/exclusion characteristics were not well defined, but there were five recent studies containing “long” or “long term” in their title.

### Long-term symptoms and effects on physical health

Table 1 summarizes current studies in the literature focused on the long physical symptoms of COVID-19 illness in the pediatric population. To date, a few studies with limited sample sizes, focusing on the non-hospitalized pediatric population, found that frequently reported physical symptoms were reported on average 3–6 months after infection including fatigue, muscle and joint pain, headache, insomnia, respiratory problems, palpitations, difficulty in concentration, and sensory problems [20, 29–32]. Weight gain due to lack of exercise and atopic dermatitis triggered potentially by a lack of exposure to sunlight and the outdoors, have also been reported [33].

**Table 1:** Studies reporting long-term physical symptoms

Authors and date	Age group	Setting	Time frame	Long symptoms	Control group	Number of participants	SARS-CoV-2 infection confirmation
Bahar et al., December 1st, 2020 [34]	Patients less than 22 y	Children’s National Hospital, DC	Retrospective **(**March 13th to June 21st, 2020)	Long-term viral shedding	None	6584	RT-PCR from nasopharyngeal swabs
Wu et al., July 1st, 2020 [35]	Newborns to 15 y	Qingdao Women and Children’s Hospital and Wuhan Children’s Hospital	Retrospective (January 20th to February 27th, 2020)	Long-term viral shedding	None	74	RT-PCR from nasopharyngeal swabs
Li et al., May 19th, 2020 [33]	Children < 14 y	Children’s Hospital, Zhejiang University School of Medicine in Zhejiang Province	Retrospective (January 1st, 2020 to March 31st, 2020)	Weight gain and atopic dermatitis	None	Not reported	Not reported
Noh et al., January 21st, 2020 [36]	Children and adolescents ≤ 19 y	Households in Northern Virginia, U.S.	Cross-sectional, observational study (July to October, 2020)	None specifically SARS-CoV-2 antibody rate was double the adult rate Robust immune response to the nucleocapsid antigen	None	1500	PCR
Buonsenso et al., January 26th, 2021 [20]	Children ≤ 18 y	Fondazione Policlinico Univeersitario A. Gemelli IRCCS (Rome, Italy)	Cross-sectional (September 1st, 2020 to January 1st, 2021)	Long-term health symptoms 120 d after COVID-19 infection, including fatigue, muscle and joint pain, headache, insomnia, respiratory problems and palpitations	None	1733	Not reported
Buonsenso et al., January 20th, 2021 [37]	Adults, and children younger than 18 y	Fondazione Policlinico Univeersitario A. Gemelli IRCCS (Rome, Italy)	Prospective cohort (May 25th to July 15th, 2020)	None specifically Similar rates of SARS-CoV-2 IgG in all age groups studied	None	110	PCR from nasopharyngeal swabs
Ludvigsson, March 2021 [38]	Children with median age of 12 y (range 9–15)	Sweden	Case report	All five children had fatigue, dyspnea, heart palpitations or chest pain, and four had headaches, difficulties concentrating, muscle weakness, dizziness and sore throats	None	5	PCR and SARS-CoV-2 antibody testing
Nogueira López et al., March 28th, 2021 [29]	Children with median age of 142 mon (IQR 117.8–166.8)	Spain	Prospective cohort (March to June 2020)	Persistent low‐grade fever, intense asthenia and severe headache	None	72	RT-PCR
Radtke et al., July 15th, 2021 [30]	Children with median age of 11 y (IQR 9–13)	55 randomly selected schools in the canton of Zurich in Switzerland	Prospective cohort (October and November 2020 to March and April 2021)	The most frequently reported symptoms lasting more than 12 wk among seropositive children were tiredness (3/109, 3%), difficulty concentrating (2/109, 2%), and increased need for sleep (2/109, 2%)	Population-based seronegative group	1355	SARS-CoV-2 antibody testing
Osmanov et al. July, 2021 [31]	Children ≤ 18 y	Z.A. Bashlyaeva Children's Municipal Clinical Hospital (Moscow, Russia)	Prospective cohort study (April 2nd, 2020 to August 26th, 2020)	Persistent symptoms among which fatigue (53, 10.7%), sleep disturbance (36, 6.9%) and sensory problems (29, 5.6%) were the most common	None	518	RT-PCR

*RT-PCR* reverse transcription-polymerase chain reaction, *SARS-CoV-2* severe acute respiratory syndrome coronavirus 2, *COVID-19* coronavirus disease 2019, *IgG* immunoglobulin G, *IQR* interquartile range

From the many studies examining COVID-19 acute symptoms, a few studies also reported on immunological findings such as long-term viral shedding, or longer duration of viral particle expulsion through daily activities such as talking, exhaling, and eating. Two retrospective studies, one from the U.S. and one from China, both reported long-term viral shedding [34, 35]. There are also many studies regarding immunoglobulin G (IgG) levels and immunological responses following COVID-19 infection. For example, one cross-sectional study found that the SARS-CoV-2 IgG rate was double in the children population compared to the adult population [36]. Conversely, another study found similar antibody rates across all age groups [37].

### Long-term effects on mental health

Studies pertaining to the long-term effects on mental health are included in Table 2. The most common mental health issues reported in the pediatric population throughout the COVID-19 pandemic were anxiety and depression, and these were only reported in papers looking at the COVID-19 pediatric patient groups [39, 40]. From two non-hospitalized cross-sectional studies in China, one examined mental health effects in primary schools and the other in junior and senior high schools, with both studies reporting anxiety and depression during home confinement during the first few months of 2020 [39, 40]. Conversely, a study in the U.S. examined the experiences of children within households identifying as Chinese–American. The authors found poorer mental health statuses as associated with higher levels of perceived racial discrimination [26].

**Table 2:** Studies reporting long-term mental health outcomes

Authors and date	Age group	Setting	Time frame	Symptoms (long), if any	Control group	Number of participants	SARS-CoV-2 infection confirmation
Li et al., January 19th, 2021 [39]	Adolescents	Junior and senior high schools in Wuhan	Cross-sectional **(**March 30th to April 7th, 2020)	Anxiety and depression	None	7890	Not reported
Xie et al., September 1st, 2020 [40]	Students grades 2–6	2 primary schools in the Hubei province	Cross-sectional **(**February 28th to March 5th, 2020)	Anxiety and depression	None	1780	Not reported
Cheah et al., November 1st, 2020 [26]	Parents, and children aged 10–18 y	Households in the U.S. that self-identified as Chinese	Retrospective cohort (March 14th to May 31st, 2020)	Higher levels of perceived racial discrimination were associated with poorer mental health	None	773	Not reported
Gassman-Pines et al., October 1st, 2020 [24]	Parents of a child or children aged 2–7 y	Large U.S. city	Prospective cohort (February 20th to April 27th, 2020)	Increase in parental reporting of daily negative moods	None	645	Not reported
Luijten et al., November 4th, 2020 [28]	Children and adolescents aged 8–18 y	Two Dutch representative samples of children and adolescents in the Netherlands	Cross-sectional Before COVID-19 (December 2017-July 2018) and during the COVID-19 lockdown (April/May 2020)	Significantly worse PROMIS T-scores on all domains Depressive symptoms, severe anxiety, and mental and health complaints	None	884	Not reported
Alves et al., October 23rd, 2020 [21]	Children aged 9–15 y	Virtual visits during “stay-at-home" measures in the U.S.	April 22nd to July 29th, 2020	Anxiety scores more than 5 standard deviations greater than values from healthy pediatric populations prior to the pandemic	None	65	Not reported

*COVID-19* coronavirus disease 2019, *PROMIS* Patient-Reported Outcome Measure Information System

The daily moods of children were more frequently reported as negative during the pandemic as compared to before [24]. However, children that engaged in more physical activity during the pandemic reported less states of anxiety [21, 41]. A non-hospitalized, cross-sectional study in the Netherlands found significantly worse Patient-Reported Outcome Measure Information System (PROMIS) T-scores on all domains, when comparing data from 2017 to 2018 to data collected during April and May 2020 [28]. Mental health effects associated with the COVID-19 pandemic included depressive symptoms, severe anxiety, and patient-specific mental and social health complaints [28].

### Long-term effects on social and behavioral health

Studies pertaining to the long-term effects on social and behavioral health are included in Table 3. For hospitalized neonates, the only long-term effects on behavioral health reported by parents were feeding, such as difficulties with or refusal to feed [23]. For older children, behavioral symptoms reported included clinginess, distraction, irritability, and fear of asking questions about the epidemic [22]. Other findings related to mood and emotional status included increases in being affectionate, restless, and frustrated [27]. The behavioral health of non-hospitalized children with COVID-19 had been reported by parents to have been worsening as the pandemic was progressing [42].

**Table 3:** Studies reporting long-term social/behavioral symptoms

Authors and date	Age group	Setting	Time frame	Symptoms (long), if any	Control group	Number of participants	SARS-CoV-2 infection confirmation
Aguilar-Farias et al., Feb 12th, 2020 [27]	Children aged 1–5 y	Households in Chile	Cross-sectional (March 30th to April 27th, 2020)	More affectionate, more restless, and more frustrated	None	1727	Not reported
Patrick et al., Oct 1st, 2020 [25]	Children aged less than 18 y	Households in U.S.	Retrospective (June 2020)	Worsening behavioral health	None	1012	Not reported
Jiao et al., April 3rd, 2020 [22]	Children aged 3–18 y	Pediatric populations in COVID-19-affected areas in China during the outbreak, specifically those in Shaanxi Province	Review of a preliminary study in Shaanxi Province during the second week of February 2020	Clinginess, distraction, irritability, and fear of asking questions about the epidemic	None	320	Not reported
Parri et al., December 1st, 2020 [23]	Children aged 0–18 y	17 Italian pediatric emergency departments	Cohort (March 3rd, 2020 to May 2nd, 2020)	For hospitalized neonates: difficulties with or refusal to feed	None	170	PCR from nasopharyngeal swabs
Luijten et al., November 4th, 2020 [28]	Children and adolescents aged 8–18 y	Two Dutch representative samples of children and adolescents in the Netherlands	Cross-sectional Before COVID-19 (December 2017-July 2018) and during the COVID-19 lockdown (April/May 2020)	Significantly worse PROMIS T-scores on all domains Worse: peer relationships, anger, sleep-related impairment, poor global health, social health complaints, effect on atmosphere at home, and negative impact of the COVID-19 regulations on daily life	None	884	Not reported
Al-Rahamneh et al., July 2021 [32]	Children ages 5–11 y	Jordan	Cross-sectional survey April 10th, 2021–April 17th, 2021	Being bored (77.5%), irritable (66%), likely to argue with the rest of the family (60.7%), nervous (54.8%), reluctant (54.2%), and lonely (52.4%) were the most frequently reported symptoms compared to the pre-COVID-19 period	None	1309	Not reported

*COVID-19* coronavirus disease 2019, *PROMIS* Patient-Reported Outcome Measure Information System

In addition, a study among adolescents aged 8–18 years in the Netherlands has reported significant worse PROMIS T-scores on all domains including peer relationships, anger, sleep-related impairment, poor global health, social health complaints, effect on atmosphere at home, and negative impacts of the COVID-19 regulations on daily life [28].

### Long-term effects on academics/child care

Although long-term effects on academic performance and learning outcomes from COVID-19 pandemic were not found in this review, a previous research suggested long-term follow-up and care of survivors from natural disaster is essential [13]. The majority of articles regarding effects on school/childcare in general, focused primarily on changes in the organizational environment (i.e., in-person, virtual) and differences in COVID-19 infection rates. The locality, setting, duration, and stage of the pandemic that the study was conducted, were all limiting factors when comparing and contrasting the studies. For example, for childcare programs, it was suggested that findings should only be interpreted within the context of transmission rates and the infection mitigation efforts implemented by each program [43]. Throughout 2020, parents reported loss of child care alongside worsening parental mental health and child behavioral health [25]. In schools, there was an increase in the lack of access to technology and internet [44]. There was also an important precedence for developing school reopening plans to protect students who are most vulnerable to learning loss or reduced access to basic needs [44].

Other studies found varying associations in transmission rates among school setting/delivery while some took into account trends before and after school re-openings. A Florida county-level study found a 1.2-fold increase in COVID-19 infection rates among elementary schools, 1.3 in high schools and no effect for virtual learning, after school re-openings [45]. Conversely, a national study in Italy 1 month after school re-openings found low transmission in schools, mainly among younger students [46]. A U.S. state-level study found an increased prevalence of COVID-19 in adolescents and youth compared to adults in the summer of 2020 [25].

### Long-term effects on quality-of-life and social determinants of health

Studies pertaining to long-term effects on quality-of-life and the social determinants of health were not outlined in a table, as these outcomes relate more to parents and changes in environmental settings. Many articles focused on examining quality-of-life issues such as nutrition, home environment, overall well-being, daily moods, and mental/emotional attitudes toward the pandemic and quarantine measures. They also analyzed effects on social determinants of health such as insurance status, healthcare needs, food insecurity, housing, income status, and caregiving burdens. These outcomes were mostly measured from parental surveys and questionnaires completed by parents or caregivers. In a non-hospital study, authors found an increase in food insecurity, nutrition barriers, homelessness or use of temporary housing [44]. Similarly, another study found an increase in moderate to severe food insecurity, alongside changes in insurance status [25]. One parent survey found an increase in frequency of parent-reported daily negative mood [24]. This article also found that the parents’ and children’s well-being was strongly associated with the number of reported hardships [24]. Hardships included job loss, income loss, caregiving burden, and illnesses.

Preliminary reports found similar findings in worsening quality-of-life issues and increased health inequities among social determinants of health. Parents experienced anxiety, changes and limitations to healthcare access, and overall “collateral” damage to their well-being as a result of economic impacts and social isolation [47]. A cross-sectional study on children and adolescents in the Netherlands found an increase in mental and social health complaints during the lockdown with the majority reporting a negative impact of COVID-19 on their life [28]. Families were concerned about the COVID-19 pandemic and quarantine measures, especially towards negative impacts on the economy [48]. These findings also raise concern regarding stability and safety within the home environment. The pandemic’s impact on child abuse and claims remains hidden, underscoring the need for further research in this field [49].

## Discussion

To the authors’ knowledge, this is one of the first scoping reviews focused on the long physical, psychological, behavioral, academic, and social consequences of COVID-19 disease and the pandemic in general in the pediatric population. From November 2019 to September 2021, our search found that out of approximately 130 publications, roughly 34 contained relevant information, and 5 specifically examined “long-hauler symptoms” or long-term effects in the pediatric population. One of the largest long-COVID-19 study was a cross-sectional study from Italy examining long COVID-19 in a small sample (*n* = 129) of children (≤ 18 years), with more than half of their patients reporting at least one long-term symptom [20]. Our search findings were also consistent with the systematic review by Ludvigsson et al., published March 2021 in Sweden. The authors reviewed 179 publications, deemed 19 relevant, and did not find any containing information on long COVID-19 in children [38]. They also included findings from their case report on five patients, who all presented with the primary persisting symptom of fatigue 6–8 months following a clinical COVID-19 diagnosis [38].

**Fig. 1:**
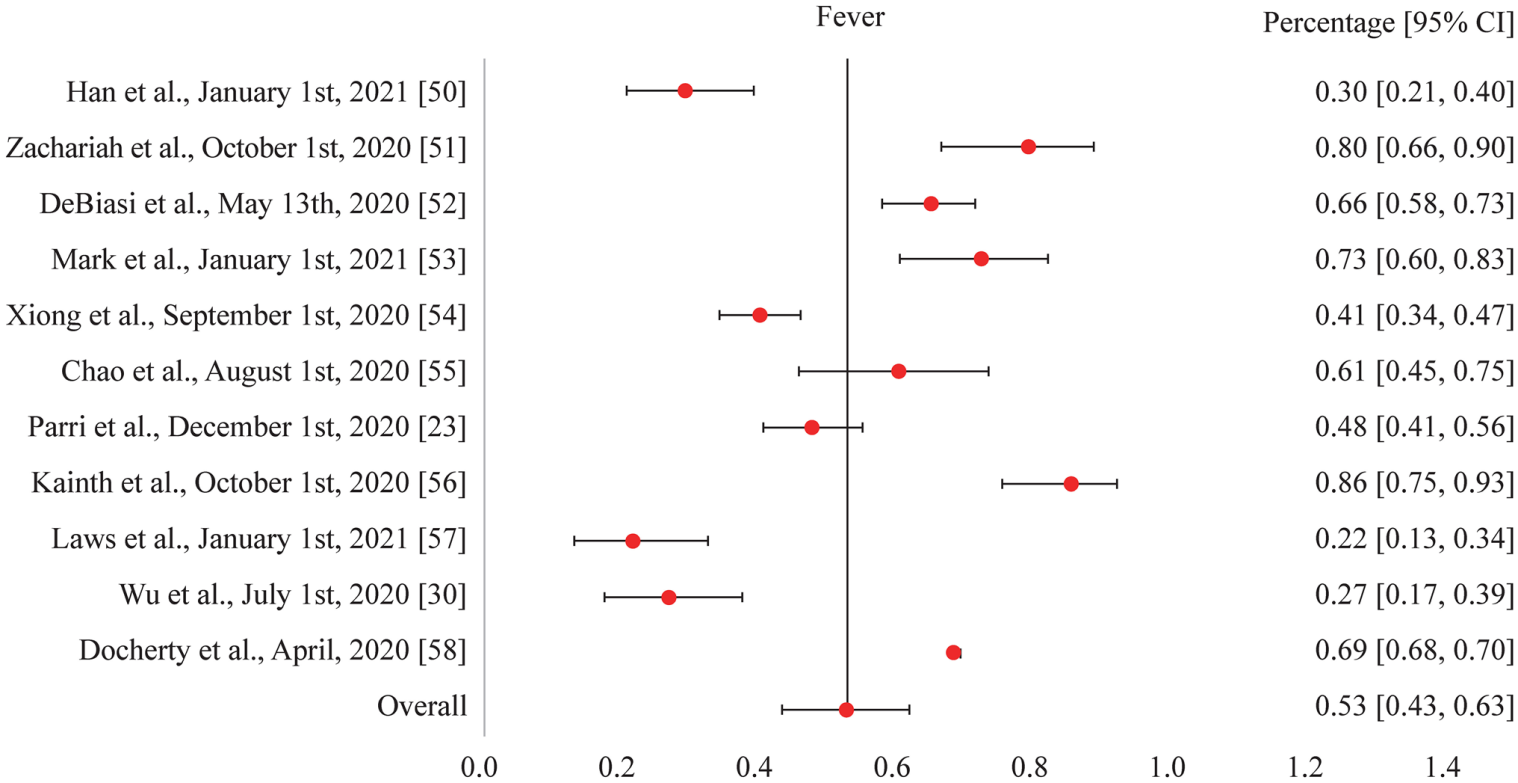
Forest plot for the acute symptom of fever from COVID-19 illness among children as reported in various studies. *COVID-19* coronavirus disease 2019, *CI* confidence interval

**Fig. 2:**
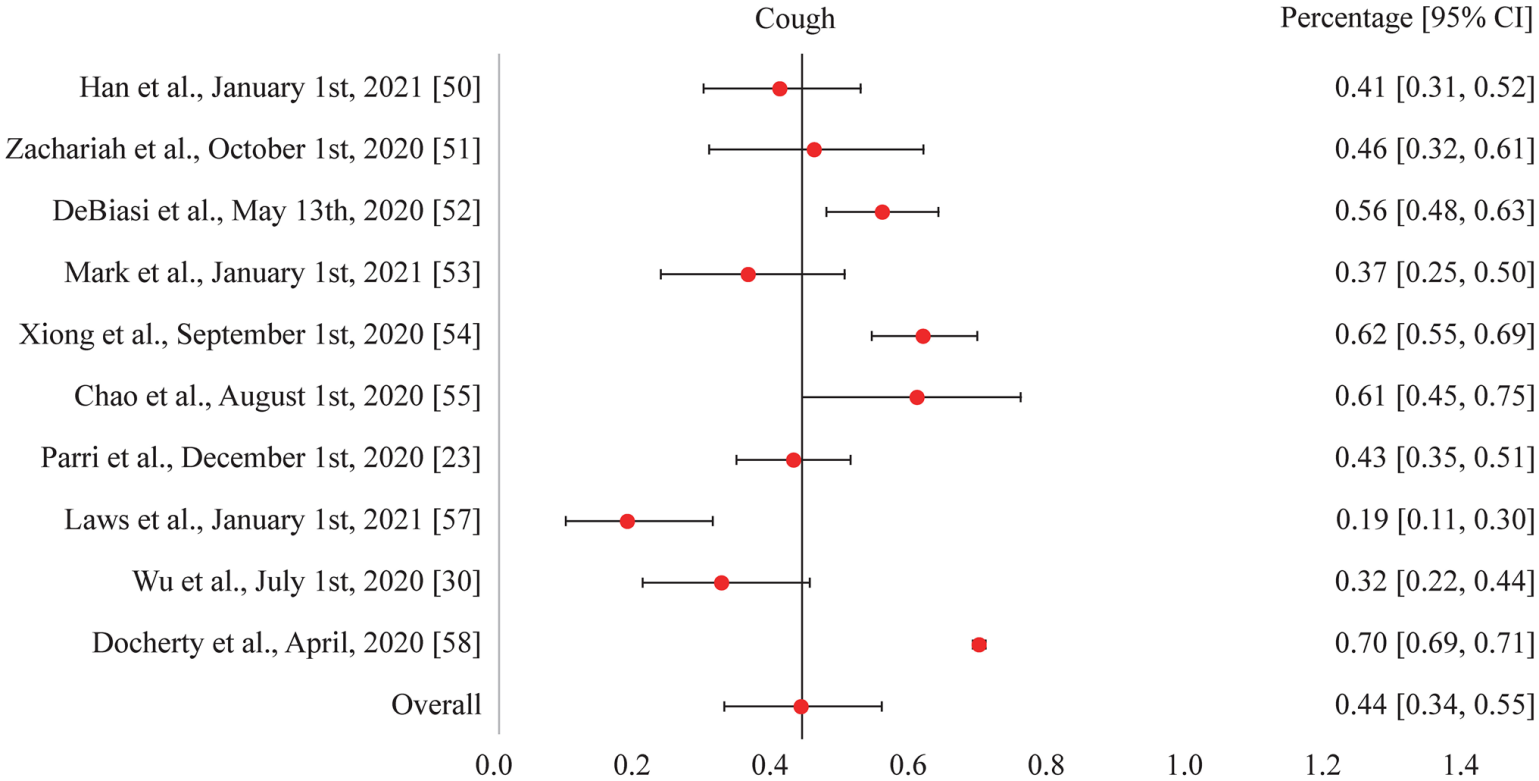
Forest plot for the acute symptom of cough from COVID-19 illness among children as reported in various studies. *COVID-19* coronavirus disease 2019, *CI* confidence interval

As of March 2021 and within the scope of our review search parameters, one study has now been published reporting physical long symptoms in children. While all the common acute symptoms of COVID-19 such as fever, cough, shortness of breath, chills, muscle pain, headache, sore throat, and loss of taste or smell were reported in the pediatric studies reviewed, the symptoms seen consistently were fever and cough (our study investigated the acute symptoms of fever and cough from COVID-19 among children, see Figs. 1 and 2). There were additional respiratory symptoms reported across all pediatric population groups, such as sputum production, along with gastrointestinal (e.g., diarrhea), cardiovascular (e.g., cyanosis), and neurological (e.g., apnea) symptoms [11, 12, 16, 35, 50–61]. Symptoms that commonly persisted following a normal infection recovery period included standard symptoms such as fever, cough, shortness of breath, muscle pain, and a headache. Some additional physical long symptoms observed in children were insomnia and heart palpitations. The persistence of these symptoms could possibly be attributed to SARS-CoV-2 triggering an abnormal immunological or inflammatory response in specific areas of the body that express the ACE2 receptor [34, 35, 62]. The invasion and persistence of SARS-CoV-2 in the central nervous system could also potentially be associated with the occurrence of mental health issues such as anxiety and depression, with further research needed to understand the mechanisms of action [63, 64]. However, many long symptoms and their etiologies may be subjective in nature and, therefore, additional research is needed to investigate the pathogenesis of long-COVID-19 symptoms across all pediatric groups, including those clinically diagnosed with MIS-C and PIMS-TS.

The majority of reviewed studies pertaining to the long-term effects of COVID-19 in children focused on mental health, social/behavioral health and environmental outcomes as a result of quarantine and social distancing. The social impact of COVID-19, primarily the mandated stay-at-home orders in 2020 and continued social distancing protocols into 2021, continues to contribute a larger role in the maintenance of social/behavioral health and mental health disorders, or at minimum, their individual symptoms. Social interaction including familial and peer relationships is integral to the development, growth and learning environment of children. The physical and emotional interactions with other individuals, both of their own age and older, facilitates proper neural development, especially regarding impulse control, mood regulation and academic development. With the potential for physical symptoms to exacerbate psychological symptoms and all child age groups awaiting COVID-19 vaccine approval, further research is needed to determine the full course of SARS-CoV-2 in the pediatric body and any persisting long-hauler effects that could compromise quality of life, even if mild in severity.

## Conclusions

In contrast to earlier reports suggesting that the outcomes or physical effects of COVID-19 in the pediatric population were milder or less severe in comparison to the adult population, the findings from this review indicate that a subgroup of children are still at risk to develop more severe and long-term presentation of symptoms, even more so for children diagnosed with MIS-C, PIMS-TS and multiple organ system failure. In addition, COVID-19 has had a moderate impact on children and adolescents’ social environment, which may exacerbate current and future physiological, psychological, behavioral, and academic outcomes. The relative lack of evidence evaluating the long-term effects of the recent COVID-19 pandemic and infections on the pediatric population, suggests the need for more prospective studies examining the long-hauler effects of an initial infection, as compared to retrospective/cross-sectional studies examining symptom presentation. This review serves as a continuum in which further research is needed to thoroughly investigate and understand the complete effects, from acute to long-term, that SARS-CoV-2 induces in the human body, especially for the pediatric population.

## Data Availability

Not applicable.
